# Exploring Resting Sinus Tachycardia in Cancer Care: A Comprehensive Review

**DOI:** 10.3390/jcm14030985

**Published:** 2025-02-04

**Authors:** Yeva Fakih, Moied Al Sakan, Alaaeddine El Ghazawi, Maurice Khoury, Marwan M. Refaat

**Affiliations:** 1 Faculty of Medicine, American University of Beirut Medical Center, Beirut 1107, Lebanon; ytf02@mail.aub.edu (Y.F.); mk04@aub.edu.lb (M.K.); 2 Internal Medicine Department, American University of Beirut Medical Center, Beirut 1107, Lebanon; ma569@aub.edu.lb (M.A.S.); aae89@mail.aub.edu (A.E.G.); 3 Cardiology Department, Division of Cardiac Electrophysiology, American University of Beirut Medical Center, Beirut 1107, Lebanon

**Keywords:** cancer, tumor, neoplasm, malignancy, leukemia, lymphoma, resting sinus tachycardia

## Abstract

Resting sinus tachycardia is frequently encountered in cancer patients. It affects a wide variety of cancer patients and is associated with distressing symptoms. Cancer-associated resting sinus tachycardia varies in its underlying mechanism. It can stem from the tumor burden or the side effects of chemotherapy/radiotherapy, or it can be secondary to paraneoplastic syndrome or the sequalae of cancer itself (infection, anemia, thrombosis, etc.). The clinical significance of resting sinus tachycardia extends beyond mere symptomatology, as it can potentially indicate severe complications which may facilitate or exacerbate a new or underlying cardiovascular dysfunction. Therefore, this necessitates thorough diagnostic tools to discern the underlying cause and tailor appropriate management strategies, whether pharmacological, non-pharmacological, or conservative. While resting sinus tachycardia has been extensively investigated in the context of cardiovascular disease, its underlying etiology, clinical implication, prognostic value, and treatment options remain vague in the context of cancer. This review aims to explore the topic of resting sinus tachycardia in cancer patients through delving deeper into its underlying mechanism, presenting the current evidence on its effect on cancer-independent cardiovascular and all-cause mortality, as well as providing some insight into the currently available treatment options. It will also propose therapeutic interventions and strategies aimed at optimizing cancer patient care. Lastly, it will highlight research gaps which need to be addressed further, as future research is needed to refine the diagnostic criteria, develop targeted therapies, find alternative cardioprotective/cardio-neutral chemotherapy options, and establish evidence-based guidelines to improve outcomes in this vulnerable patient population.

## 1. Introduction

Cancer and cardiovascular disease (CVD) are the leading causes of death worldwide. Both share several common risk factors, which include older age, poor diet, sedentary lifestyle, elevated BMI, pollution, smoking, and alcohol [1,2]. Emerging research suggests that chronic inflammation may have a shared pathological mechanism linking these two conditions [3]. In oncology patients, the development of cardiovascular dysfunction extends beyond the well-documented cardiotoxicity of chemotherapy and radiation. These include arrhythmias and sinus tachycardia, as well as hemodynamic instability, atherosclerosis, and other cardiovascular complications [4]. Understanding these processes allows for earlier detection of cardiovascular compromise and establishment of better prevention strategies.

Elevated resting heart rate (HR) was evident to be an independent risk factor for all-cause mortality rates [5,6]. An increase in resting HR was linked to numerous cardiovascular and metabolic diseases [7,8,9]. The Framingham study showed that for every increase of approximately 10 beats per minute (bpm) in HR, there is a consequent 14% escalation in mortality [6]. In addition, case fatality rates, sudden death from a coronary cause, and non-cardiovascular and cardiovascular deaths all increased with elevated resting HR [6].

Another study in patients with stable CVD showed that resting and average HR are related to increase in all-cause mortality and cardiovascular events [7]. Additionally, for every increase in resting HR by 1 bpm, there was a subsequent 4% jump in relative risk (RR) of incident heart failure (HF) [8]. Such an increase affected left ventricular ejection fraction (LVEF) with a subsequent quantitative decrease in circumferential strain (εcc) due to impaired circumferential fiber contraction, a phenomenon often seen in cases of HF or cardiomyopathy [8]. In simple terms, myocardial strain refers to the extent of myocardial muscle deformation as the heart beats. Circumferential strain, in particular, refers to the stretching and shortening of myocardial fibers along a circular axis around the heart (LV walls), with negative strain indicating stretching and positive strain indicating shortening. The aforementioned parameter remains a key component in assessing early changes in myocardial and left ventricular function. So, not only is elevated baseline HR an independent contributor to HF, but it is also one of the earliest hemodynamic markers of left ventricular dysfunction (LVD), even before the onset of clinical symptoms or radiological wall-motion abnormalities [8]. Plus, a target HR of less than 80 bpm showed a benefit in patients with HF, despite that the mechanism remains vague [10].

Alternatively, elevated resting HR has been associated with HF and LVD even in asymptomatic patients with no previously known CVD. Since cardiac output (CO) is dependent on two major parameters, stroke volume (SV) and HR, a minor decrease in SV due to clinically insignificant LVD could potentially trigger a compensatory elevation in baseline HR [11]. Such an increase occurs before any neuroendocrine activation (norepinephrine) is identified [12].

With the prognostic value of elevated resting/baseline HR being established in the general population and in patients with HF or CVD, this review aims to better understand the association between resting sinus tachycardia and adverse cardiovascular outcomes (ACVOs), as well as survival in cancer patients. Specifically, resting sinus tachycardia could be an early sign of cardiovascular stress and compromise [13]. Thus, establishing its prognostic value could be the first step towards better management and hence prolonged survival in cancer patients [13].

## 2. Methodology

Three databases, including Pubmed, Ovid Medline, and CINAHL, were searched until 3 October 2024 using both keywords and MeSH terms, including “Cancer” OR “Tumor” OR “Neoplasm” OR “Malignan*” OR “Leukemia” OR “Lymphoma” and “Sinus Tachycardia”. The outcome of interest, “Adverse Cardiovascular Outcome”, was not included in the search strategy due to a very low number of papers. The articles included retrospective and prospective case–control and observational studies which discussed the prognostic value of elevated baseline HR or ECG-determined resting sinus tachycardia on ACVOs and survival in cancer patients. Exclusion criteria included articles which tackled the overall prognosis and complications of specific cancer subtypes.

Two authors screened the titles of relevant articles and abstracts. The yielded articles were screened to fit the inclusion/exclusion criteria and duplicates were removed. In case of any disagreement regarding the relevancy of an article, the two authors discussed the subject matter until consensus was reached. Since the search yielded a very low number of articles, the authors manually screened the reference lists of those articles in order to identify additional relevant papers.

## 3. Hemodynamic Variability in Cancer

Cancer patients notably suffer from hemodynamic dysfunction. SV, CO, systolic blood pressure (SBP), and baseline HR are significantly higher in cancer patients as compared to the general population [14]. Additionally, research showed that during isovolumetric contraction (early systole), the pressure rise at a maximal rate is higher in cancer patients compared to the general population, which equates to greater strain on the heart [14]. It has been hypothesized that the aforementioned parameters increase due to the elevated metabolic demand and sympathetic overdrive, which are related to tumor burden [13]. Consequently, it sheds light onto the presence of an elevated cardiovascular risk in this vulnerable group of patients as compared to the general population, which undoubtedly requires more investigation.

Geigar et al. studied the changes in CO through a Doppler-ultrasound technique, as compared to the widely used echocardiography, in patients with several types of cancer taking an anthracycline-based regimen [15]. The study showed that chemotherapy causes an initial transient increase in CO followed by a permanent decrease [15]. Trimarchi et al. mentioned that global longitudinal strain (GLS), introduced recently, is a more effective measure than LVEF in detecting and handling cancer treatment-associated cardiac dysfunction [16]. Of note, anthracyclines have been widely associated with severe cardiomyopathies due to their prominent cardiotoxic effect [17]. Another study aimed to assess the autonomic dysregulation (AD) seen in cancer patients due to various etiologies, such as chemotherapy, poor lifestyle, radiation exposure, and the cancer itself [18]. The study showed that components of AD, which includes vagal–sympathetic malfunction, increased resting HR, impaired HR recovery, and minimal variability in HR, all serve as potential prognostic factors for survival in various cancer subtypes [18]. Also, the hemodynamic instability seen in cancer patients has been directly related to the future development of hypertension, ischemia, arrythmias and heart failure. In summary, cancer is a strong multi-system stressor which puts significant strain on normal physiology and hemodynamics.

### 3.1. Sinus Tachycardia in Cancer

Elevation in HR originating from the sinoatrial (SA) node, termed sinus tachycardia, is a frequent cardiovascular phenomenon seen in many cancer patients [14,17]. In order to grasp the complex interplay between cancer, cancer treatments, and cardiovascular health, the normal physiology of HR control should be outlined first. It is essential to differentiate resting sinus tachycardia from other tachyarrhythmias, with the former characterized by HR > 100 bpm originating from the SA node. Diagnosis involves ECG, Holter monitoring, echocardiography, autonomic function tests, and biomarkers (CRP, cytokines, troponin).

HR is carefully regulated by the autonomic (involuntary) nervous system, which includes the sympathetic and parasympathetic systems [19,20]. The right atrium houses the SA node, which serves as the innate pacemaker of the heart [21,22]. The electric signal originates at a constant frequency in the SA node, which ultimately sets the rhythm of the heartbeat, a phenomenon described as “autorhythmicity” [22]. HR is cautiously regulated to respond adequately to the various human body needs and thus changes during rest, walking, exercise, stress, etc. [23]. Such regulation is governed by a neuroendocrine response to a change in our usual state of hemostasis. The parasympathetic system decreases the HR through increasing vagus nerve simulation, while the sympathetic system increases the HR through a catecholamine surge, thus responding sufficiently to any earthly requirement [20,22].

The mechanism of resting sinus tachycardia in cancer patients is due to several factors that disrupt the normal regulation of the HR. Examples include tumor burden, chemotherapy, radiotherapy, and lifestyle factors [24]. One prospective observational study showed that cancer patients have an elevated resting HR regardless of treatment initiation [25]. This shows that cancer itself holds a substantial detrimental effect on cardiovascular health beyond the well-known cardiotoxicity seen with certain cancer treatments [24].

Cancer triggers inflammation, elevating inflammatory markers and activating the sympathetic nervous system and thus raising baseline HR [24,26]. Sakellakis et al. highlight cancer-induced resting sinus tachycardia as a common but often overlooked condition, driven by structural cardiac changes, inflammation, cytokine release, thromboembolism, compensatory responses, and pain [24].

Additionally, it is quite common that cancer patients experience anxiety, depression, and increased levels of stress [27]. Stress is well known to tip the scale towards elevated HR. The sympathetic-to-parasympathetic ratio increase seen with stress is directly related to increased mortality in the general population, with its extrapolation being feasible to cancer patients as well [28].

Chemotherapy agents, including anthracyclines, alkylating agents, antimetabolites, and TKIs, are highly cardiotoxic [29]. Anthracyclines, in particular, damage cardiac myocytes, reducing CO and triggering compensatory mechanisms that raise HR [17]. Chemotherapy also induces systemic inflammation and cytokine release, further activating the sympathetic nervous system and increasing baseline HR [30,31].

Radiotherapy damages the pericardium, coronary vessels, cardiac myocytes, and the SA node, leading to atherosclerosis, fibrosis, and autonomic dysfunction, which can cause resting sinus tachycardia [32,33,34]. Radiation-induced inflammation also contributes to elevated HR [31]. Immunotherapy, such as immune checkpoint inhibitors, causes myocarditis, cardiomyopathy, and cytokine release syndrome, all of which strain the heart and increase HR [35,36,37]. Additionally, post-HSCT autonomic dysfunction and inflammation raise HR further [38].

**Figure 1:**
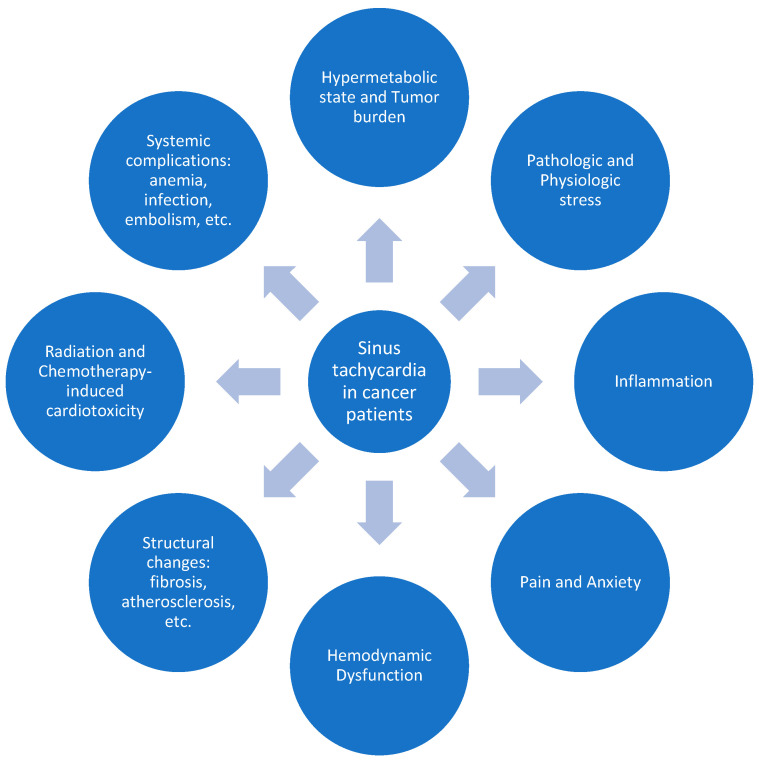
Summary of key contributors to resting sinus tachycardia in cancer patients.

The combined impact of various cancer therapies, tumor burden, lifestyle and myocardial structural changes, and systemic inflammation disrupts autonomic balance, increasing sympathetic activity and contributing to resting sinus tachycardia (Figure 1). Timely identification and management are crucial for improving outcomes and survival.

### 3.2. Association Between Resting Sinus Tachycardia and Cardiovascular Outcomes in Cancer Patients

Elevated resting HR is a known predictor of cardiovascular mortality in both the general population and individuals with chronic CVD [6,7,8]. Multiple studies have shown the effect of tachycardia on cardiac hemodynamics, such as higher LV filling pressures, greater LV wall tension, and increased systemic resistance [39]. However, research specifically on resting sinus tachycardia is limited, especially in cancer patients (Table 1). To date, only one study has examined the link between resting sinus tachycardia and ACVOs in this population [13].

A study by Hemu et al. included 622 patients: 51 cancer patients with ECG-defined resting sinus tachycardia age- and sex-matched to 571 controls [13]. It looked at several cardiovascular parameters, including HF, and showed that resting sinus tachycardia is associated with an increased incidence of HF with reduced ejection fraction (HFrEF) and acute HF exacerbation (AHFE) but not HF with preserved ejection fraction (HFpEF) [13]. The reason could be due to a steady rise in myocardial oxygen demand because of a constantly elevated HR. That can ultimately worsen a pre-existent cardiac compromise or ignite new-onset HF. The study also reiterated that resting sinus tachycardia is associated with an increased risk of myocardial infarction (MI), arrhythmias, cardiomyopathy, thromboembolic events, and death [13].

### 3.3. Association Between Elevated Resting Heart Rate/Sinus Tachycardia and Overall Survival in Cancer Patients

The prognostic value of resting sinus tachycardia has been well established in the general population [5]. More recent studies have confirmed its vital role in predicting cardiovascular outcomes and survival in cancer patients (Table 1). The study conducted by Hemu et al. showed that resting sinus tachycardia is an independent predictor of mortality in cancer patients after adjusting for confounders [13]. However, this study was limited by a small sample size, discrepancy between the number of cases and controls (51 vs. 571), a small Caucasian subset in the case group, and ambiguity in regard to the presence of resting sinus tachycardia prior to cancer diagnosis.

Another prospective case–control study, conducted between 2005 and 2010, included patients with colorectal, pancreatic, and non-small-cell lung carcinoma and showed that an elevated resting HR on ECG is an independent predictor of survival in cancer patients [40]. The study conducted by Anker et al. followed 145 cases and 59 controls for 27 months and showed that healthy subjects had a statistically significant lower baseline HR on ECG as compared to cancer patients [40]. Consequently, a multivariate model showed that a resting HR ≥ 75 bpm markedly predicted survival in cancer patients [40]. The results of the study suggest that elevated resting HR could serve as an important marker for survival in cancer patients. However, the limitations of the study include an inability to precisely determine the cause of death due an overly complex clinical picture in a high-risk patient cohort, short follow-up period of 27 months, and possibly missed confounders.

Another retrospective observational study aimed to follow up 4786 breast cancer patients, stages I-III, for a total period of 5.0 ± 2.5 years [41]. The findings revealed a significant association between higher resting HR and increased mortality [41]. Specifically, patients with a resting HR of 85 compared to those with 67 had a higher all-cause and breast cancer-specific mortality [41]. Each 10 bpm increase in HR corresponded to a 15% increase in all-cause mortality and a 22% rise in cancer-specific mortality [41]. However, Lee et al. mentioned that the precise underlying mechanism linking resting HR to cancer prognosis remains vague. It is undoubtedly a reflection of many lifestyle and behavioral elements, such as physical activity, alcohol consumption, and smoking [41]. Additionally, detailed medication history, severity of comorbidities, and pre-existing diseases were hard to entirely account for [41]. Lastly, the external validity of the study is debatable since it was geographically limited to South Korea [41]. Hence, further research should include a more diverse cancer patient population.

Similarly, a prospective observational study followed up 104 lung cancer patients and showed that a heart rate > 90 bpm on ECG is an independent negative predictor of survival [42]. The study proposed that incorporating HR monitoring into routine clinical assessment could improve prognostic accuracy and influence management options. However, the result was not statistically significant, so one might consider this study with caution. Another study was conducted at the Rush University Medical Center and involved 622 cancer patients with various types of cancer, including lung cancer, lymphoma, leukemia, and multiple myeloma over 8 years [43]. It showed that tachycardia was an independent and significant predictor for all-cause mortality [43]. The main power-limiting setback of the study is its small sample size (50).

Furthermore, one prospective study in 548 unselected treatment-naïve cancer patients showed that for each 5 bpm increase in resting HR, there was an increase in all-cause mortality, even after adjusting for other variables such as tumor stage and cardiac health at presentation [25]. Also, researchers performed a subgroup analysis differentiating between the types of cancer. Consequently, elevated resting HR was most strongly associated with mortality in lung and gastrointestinal cancer [25]. The major drawback of the study includes a single baseline HR measurement prior to cancer treatment initiation, rather than multiple measurements on several visits or continuous measurements with a 24 h Holter. This does not provide any insight into the average HR and HR variability and makes relying on a single measurement insufficient to draw any firm and safe conclusions.

Lastly, one study aimed to understand the clinical role of HR in post-HSCT. Since many post-HSCT patients die from CVD-related complications, whether acutely or chronically, it was crucial to delineate the possible prognostic factors which could potentially predict the risk of developing HF or arrythmias in this subset of patients [38]. Lopez-Candales et al. described that an elevated HR was associated with worse clinical outcomes, suggesting that it could serve as a prominent prognostic marker in this specific patient population [38]. This study further extends our understanding of the significance of HR assessment across different cancer treatments.

## 4. Therapeutic Opportunities

### 4.1. General Therapeutic Approaches for Managing Sinus Tachycardia

Treating resting sinus tachycardia requires a personalized approach depending on whether the patient is symptomatic or not. The symptoms of resting sinus tachycardia include palpitations, dizziness, chest discomfort, shortness of breath, anxiety or feeling of restlessness, decreased exercise tolerance, and reduced quality of life [44,45]. Treating it entails maximizing both pharmacological and non-pharmacological modalities.

The two mainstay treatment options for resting sinus tachycardia are beta blockers (BBs) and Ivabradine. The latter is more commonly used when there is poor response or intolerability to BBs due to side effects [46]. BBs reduce HR by blocking beta-adrenergic receptors in the heart [47,48]. Alternatively, Ivabradine inhibits the pacemaker I_f_ current, which is found in the SA node, thus directly reducing HR without affecting other hemodynamic variables, like in the case of BBs or calcium channel blockers (CCBs) [46,48,49]. The selective inhibition seen with Ivabradine could be the solution when the physician aims to avoid the effects of BBs or CCBs on contractility and blood pressure [49]. The effectiveness of Ivabradine in managing resting sinus tachycardia has been described in cases of tachycardia-induced cardiomyopathy, inappropriate sinus tachycardia, tachycardia-induced HF and severe cardiomyopathy with mitral regurgitation, acute HF in inflammatory rheumatic disease, and advanced HF patients on extracorporeal life support [50,51,52,53,54,55,56].

Non-pharmacological interventions are considered in conjunction with pharmacotherapy [45,57]. Examples include avoiding excessive caffeine or alcohol, hot environments, prolonged standing, dehydration, or stress [45,57] and simply avoiding anything which decreases CO or activates the sympathetic nervous system. It also entails addressing the possible underlying causes, such as hyperthyroidism, inflammation, infection, or anemia [45,50,55], and avoiding volume-depleting and veno-dilating agents such as diuretics and nitrates, which is not always possible, especially in cases of concomitant HF [57]. As stated, a huge portion of treating and optimizing elevated HR relies on internalizing a healthy lifestyle. Now, when both pharmacotherapy and non-pharmacotherapy are not enough for achieving symptomatic relief, more invasive techniques such as catheter ablation can be utilized in the treatment of resistant resting sinus tachycardia [58].

In regard to asymptomatic patients who exhibit elevated HR without experiencing any debilitating symptoms, a more conservative approach is usually desirable. It relies on lifestyle modifications and frequent follow-ups to assess the development of tachycardia-induced complications and prevent them prior to their clinical onset.

In brief, intervening is only justifiable if there are debilitating symptoms or when there is a prominent risk of progression to a more serious cardiovascular condition such as HF, arrythmias, or cardiomyopathy.

### 4.2. Cancer-Specific Therapeutic Approaches for Managing Resting Sinus Tachycardia

A personalized approach is necessary when managing such delicate patients. This should focus on the specific cancer subtype, chemotherapy/radiotherapy regimen, and individual patient characteristics.

Lai et al. showed that BBs, specifically metoprolol, can be used in treating resting sinus tachycardia peri-operatively in patients undergoing esophageal cancer resection [59]. This result could potentially be extrapolated and applied to treating resting sinus tachycardia in esophageal cancer and cancer patients in general. Alternatively, one study showed that Ivabradine can be useful in post-HSCT patients with acute HF and resting sinus tachycardia [60]. This was further supported by another study which showed favorable effect of Ivabradine in treating resting sinus tachycardia in patients suffering from paraganglioma [61]. Also, Harada et al. showed that Ivabradine is safe and efficacious in achieving HR control in breast cancer patients undergoing chemotherapy [62].

The selectivity of the mechanism of action seen with Ivabradine is especially useful in cancer patients who already have compromised cardiovascular status and hemodynamic instability [60,61,62]. On the other hand, less selective drugs such as BBs can lead to a reduction in myocardial contractility and subsequent hypotension, which could be detrimental in the setting of cancer [60,61].

While some studies suggest Ivabradine may help manage resting sinus tachycardia in cancer patients, they are limited by small sample sizes, specific populations, short follow-up duration, retrospective or case report designs, and single-centered approaches. These gaps underscore the need for larger, well-designed RCTs that assess long-term outcomes across cancer populations while accounting for confounders. However, research is challenging due to Ivabradine’s interactions with some chemotherapy agents and prophylactic antifungal drugs. For example, coadministration with voriconazole (VFEND) can cause long QT syndrome and life-threatening arrhythmias such as Torsade de Pointes [63], raising ethical concerns and limiting RCT eligibility.

Besides using pharmacotherapy solely for managing resting sinus tachycardia, there are some cardioprotective medications which can be utilized to mitigate chemotherapy-induced cardiotoxicity. Various BBs have antioxidant properties which reduce free radical formation, mitochondrial dysfunction, and lipid peroxidation, thus lowering the level of Troponin I and cardiac dysfunction [64]. Renin–angiotensin inhibitors, angiotensin receptors–neprilysin inhibitors, and mineralocorticoid receptor antagonists are well known for limiting cardiac remodeling and reducing myocardial fibrosis, with some studies showing positive effects on preserving EF during chemotherapy [64]. Additionally, the MANTICORE 101- breast trial showed promising results when using combination therapy of perindopril and bisoprolol in LVEF decline during trastuzumab therapy [65]. Statins also help through their antioxidant and anti-inflammatory properties [64]. Lastly, Dexrazoxane is the only FDA-approved compound for reducing doxorubin-induced cardiotoxicity [64]. It acts through lowering anthracycline–iron complex formation, therefore reducing reactive oxygen species (ROS) formation and consequent oxidative damage [64]. Despite this evidence, there are no accepted and universal guidelines and their addition is therefore solely dependent on patient-specific parameters and the treating physicians.

Cancer patients can also benefit substantially from addressing risk factors such as hypertension, hyperlipidemia, diabetes, smoking, obesity, and lack of physical activity before and during cancer treatment [64,66]. In particular, exercise was shown to be cardioprotective for cancer patients as it improves the tolerance of the myocardium against cardiotoxic agents, lowers the formation of ROS, and enhances overall endothelial function [64]. More specifically, endurance training reduced the cytotoxic effect of doxorubicin in vivo [67]. It was hypothesized to be due to enhancing the cellular and mitochondrial defense mechanisms and reducing oxidative stress by lowering the overall intracellular levels of doxorubicin [67]. This is quite important as doxorubicin remains one of the most cardiotoxic agents used.

Lastly, a more conservative approach entails reducing the dose of the chemotherapy agent used or administering it with a slower infusion rate [64]. For example, Van Dalen et al. showed that administering anthracyclines with an infusion duration of more than six hours was favorable in decreasing the risk of HF [68]. This is consistent with other available evidence which shows that anthracyclines exhibit a dose-dependent cellular toxicity through altering the mitochondrial membrane permeability in a dose-dependent manner [69,70,71].

Alternatively, there are newer agents that are considered more cardioprotective than the traditional formulations. Liposomal anthracycline, particularly pegylated liposomal doxorubicin, showed decreased cardiotoxicity and fewer cardiac events without compromising efficacy as compared to traditional doxorubicin [72]. But its long-term effect on cardiovascular health is still unknown [72]. Liposomal formulations are well known to concentrate in pathological areas such as in inflamed or damaged tissues. This facilitates targeted drug delivery and minimizes exposure of healthy non-targeted organs to the drug. However, in the context of a pre-existing or new “damaged” heart, one can hypothesize that such a molecule would paradoxically worsen the damage. Other potential benefits of liposomal formulations stem from their stability and resistance to degradation which enhances their bioavailability in the targeted place. Additionally, their sustained release and increased circulation time provide a stable and safer concentration for a longer period. Despite the numerous advantages, more data are needed to adequately describe their cardiovascular safety profile.

**Figure 2:**
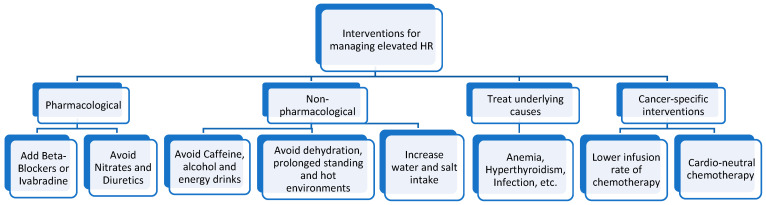
Summary of key pharmacologic and non-pharmacologic interventions for reducing HR in cancer patients.

Other agents, such as targeted therapy (e.g., monoclonal antibodies) and immunotherapy, are more selective in destroying cancerous cells and avoiding the heart, which paves the way for creating more selective cancer treatment options that will not sacrifice the heart alongside the malignancy. Figure 2 summarizes the key pharmacologic and non-pharmacologic interventions for reducing HR in cancer patients.

## 5. Knowledge Gaps and Further Research

Studies evaluating the association between elevated resting HR and the risk of ACVOs in cancer patients are scarce [38,39,40]. Most studies have concentrated on specific cancer types, namely lung, breast, colorectal, and pancreatic cancers, due to their high prevalence and associated mortality rates [38,39,40]. Only one study analyzed ECG-detected resting sinus tachycardia cumulatively in all types of cancer and described specific ACVOs [13]. Unfortunately, most studies mentioned the difficulty in addressing the precise cause of death in cancer patients due the complexity of the clinical picture and the rarity of autopsies performed, since the majority often die at home or in hospice care [24,39]. Some patients with cancer and chemotherapy-induced severe cardiomyopathy have increased mortality if they do not improve on guideline-directed medical therapy and do not have an implantable cardioverter defibrillator [73]. Further, some arrhythmias including premature ventricular contractions can lead to ASCOs [74]. Consequently, it would be hard to predict the true impact of elevated HR on the development of ACVOs, as many patients die before ACVOs manifest, or the cause of death might be due to a cardiovascular deficit which would not be captured post-mortem.

Also, it is a possibility that the resting sinus tachycardia observed in cancer patients could be a byproduct of the tumor burden, whether it be from the widespread systemic inflammation or the hypermetabolic state caused by the cancer itself. This poses a serious question of whether treating resting sinus tachycardia in this patient population would mute the compensatory response intentionally generated by the body. On the other hand, Anker et al. found that resting heart rate was not related to cancer stage; hence, terminal disease is not a prerequisite for resting sinus tachycardia [24,38]. Also, no research is available to suggest whether asymptomatic resting sinus tachycardia should be treated, besides advising for the standard life-style modifications. And no studies are available to suggest whether treating asymptomatic tachycardia would affect morbidity and mortality. The aforementioned is not described for the general CVD population, let alone cancer patients.

Lastly, cancer patients are a vulnerable population with an already increased risk of cardiovascular compromise, so addressing cardiovascular parameters here cannot just rely on studies extrapolated from the general population. Hence, this subject is a prominent gap in the literature with a crucial need for further investigation.

## 6. Conclusions

Resting sinus tachycardia is a complex phenomenon with a multifactorial etiology and prominent clinical and prognostic implications on cancer patient health and survival. It is a real clinical challenge and therefore necessitates comprehensive diagnostic approaches and treatment guidelines. It can stem from the effect of the tumor burden, wide-spread inflammation, treatment-related toxicity, comorbidities, and psychological stress. Adequate management involves utilizing both pharmacotherapy and non-pharmacotherapy, managing underlying conditions, switching or adjusting cancer treatments to involve more cardio-neutral formulations, and optimizing the quality of life, with a patient-centered and holistic multidisciplinary approach. This review focused on providing a scope of the available evidence on resting sinus tachycardia in cancer patients while focusing on its impact on ACVOs and overall survival, as well as providing a basic understanding of the accessible therapeutic options. However, it is crucial to have new and ongoing research to better grasp the pathophysiology and thus develop more targeted therapeutic strategies beyond the mainstream BBs. Establishing evidence-based guidelines to treat resting sinus tachycardia in cancer patients is one step closer towards achieving better clinical outcomes and optimizing cancer care.

## Figures and Tables

**Table 1:** Summary of key findings on the prognostic value of resting heart rate in cancer patients.

Study	Study Type	Objective	Cancer Type	Participants Number	Follow Up Period	Key Findings	Year
Hemu et al. [13]	**Prospective case–control**	Association between resting sinus tachycardia and adverse cardiovascular outcomes and mortality in cancer patients	Cancer patients	**622**	2008–2016	Resting sinus tachycardia associated with adverse cardiovascular outcomes and increased mortality	**2021**
Anker et al. [25]	**Prospective observational study **	Impact of increased resting heart rate on prognosis in treatment-naïve cancer patients	Treatment-naïve unselected cancer patients	**548**	2011–2013	Increased resting heart rate correlates with worse prognosis	**2020**
Lopez-Candales et al. [38]	**Editorial**	Clinical relevance of heart rate in post-hematopoietic stem cell transplant patients	Post-hematopoietic stem cell transplant patients			Heart rate may have clinical significance in post-hematopoietic stem cell transplant patients	**2022**
Anker et al. [40]	**Prospective case–control**	Predictive value of resting heart rate on death in cancer patients	Colorectal, pancreatic, and non-small-cell lung cancer patients	**204**	2005–2010	Resting heart rate is an independent predictor of death	**2016**
Lee et al. [41]	**Retrospective observational study**	Resting heart rate as a prognostic factor for mortality in breast cancer patients	Breast cancer patients	**4786**	5.0 ± 2.5 years	Higher resting heart rate associated with increased mortality	**2016**
Medrek & Szmit [42]	**Prospective observational study**	Baseline ECG and echocardiographic assessments for predicting survival in lung cancer patients	Lung cancer patients	**104**	3 years	ECG, echocardiographic, and RHR assessments may help predict survival	**2022**
Katie Glen [43]	**Retrospective cohort study**	Association between sinus tachycardia and mortality in cancer	Lung cancer, leukemia, lymphoma or multiple myeloma	**622**	2008–2016	Higher mortality rate in cancer patients with tachycardia	**2019**
